# MiR-7 inhibits progression of hepatocarcinoma by targeting KLF-4 and promises a novel diagnostic biomarker

**DOI:** 10.1186/s12935-017-0386-x

**Published:** 2017-02-21

**Authors:** Weizhong Wu, Sanguang Liu, Yunfei Liang, Zegao Zhou, Xueqing Liu

**Affiliations:** 1 Department of General Surgery, The Second Hospital of Hebei Medical University, Shijiazhuang, 050000 Hebei China; 2 Department of Hepatobiliary Surgery, The Second Hospital of Hebei Medical University, Shijiazhuang, 050000 Hebei China

**Keywords:** Hepatocellular carcinoma (HCC), miR-7, Diagnosis, Biomarker, Proliferation, Invasion

## Abstract

**Background:**

MicroRNAs are 22–24 nt non-coding RNAs that bind to the 3′ UTR of target mRNAs, thereby inducing mRNA degradation or inhibiting mRNA translation. Due to their implication in the regulation of post-transcriptional processes, the role of miRNAs in hepatocellular carcinoma (HCC) has been extensively studied. However, the function of miR-7 in HCC remains to be demonstrated.

**Methods:**

50 paired HCC tissues and matched peritumor tissues from patients were collected. The mRNA level of miR-7 was detected by qRT-PCR. The protein level of Kruppel-like factor 4 (KLF-4) was determined by western blot. Cell proliferation and invasive ability were measured using MTT and transwell invasion assay, respectively.

**Results:**

We demonstrated that miR-7 was downregulated in 50 HCC tissues and the low expression of miR-7 was significantly correlate with tumour size. Moreover, overexpression of miR-7 significantly inhibited the proliferation and invasion of HCC cells. Over 100 target genes of miR-7 were predicted by Targetscan, and KLF-4 was indicated as the most promising candidate. Luciferase report assay showed that KLF-4 could be silenced by miR-7, so as to restore the impairment of cell proliferation and invasion in HCC cells.

**Conclusions:**

In summary, we revealed a role of miR-7-KLF-4 axis in HCC cells, and the combination of both biomarkers might improve HCC diagnosis.

## Background

Hepatocellular carcinoma (HCC) is the fifth highest incidence rate of malignant tumor and the third leading cause of cancer-related death in the world [1]. The genetic or epigenetic regulations contributing to HCC have been well-characterized; however, the molecular mechanisms underlying pathogenesis still need to be defined [2]. MicroRNAs (miRNAs) are a class of small, non-coding RNAs, which modulate gene expression at the post-transcriptional level [3, 4]. A number of literatures reported that the aberrant expression of miRNAs is a significant feature in various cancers [5, 6]. Functional studies have indicated that miRNAs play diverse roles in tumorigenesis, such as tumor suppressors or oncogenes, and act as modulators for tumor cell survival, apoptosis, and metastasis [7]. MiR-7 is an ancient miRNA, whose mature sequence is conserved from Annelida to human. Previous studies have implicated that miR-7 is involved in the development of different types of human cancers, including breast cancer [8] and HCC [9]. In these cancers, MiR-7 targets several proto-oncogenes, such as insulin receptor substrate 1 (IRS1), epidermal growth factor receptor (EGFR), and p21 protein (Cdc42/Rac)-activated kinase 1 (PAK1) [10, 11]. In addition, Zhao et al. showed that miR-7 might function as a tumor suppressor, which inhibits cell growth and migration in non-small cell lung cancer cells [12, 13]. It has been reported that miR-7 was downregulated in HCC, but the regulatory mechanism in hepatoma cells remains unclear [14]. In the present study, we investigated the expression level of miR-7 in HCC tissues and its pathways involved in HCC cells, thus to explore new targets as the prediction index of HCC.

## Methods

### Human tissues

A total of 50 paired surgically resected HCC tissues and matched peritumor tissues were collected from patients during operation in our hospital (10 cases of T1N0M0, 13 cases of T2N0M0, and 27 cases of T3N0M1). Exclusion criteria: HBV and HCV infection HCC patients. All tissues and tumor samples were flash-frozen in liquid nitrogen immediately after collection and stored at −80 °C for future use. This protocol was approved by the Ethics Committee of The Second Hospital of Hebei Medical University, and the written informed consents were obtained from all participants prior to their participation in the study.

### Cell culture and transfection

Hepatocellular carcinoma Hep3B, SMMC-7221, and HepG2 cell lines were purchased from ATCC (Manassas, VA) and cultured in DMEM medium (Gibco, Grand Island, NY) supplemented with 10% heat-inactivated FBS, 100 U/mL penicillin, and 100 μg/mL streptomycin at 37 °C in a humidified chamber with 5% CO2. Scrambled control or miR-7 antagomir/mimic (ThermoFisher, Ambion) were transfected into cells with the Lipofectamine 2000 reagent (Invitrogen, Carlsbad, CA), following the manufacturer’s instructions.

### RNA isolation and quantitative RT-PCR (qRT-PCR)

Total miRNAs from liver tissues and cell lines were isolated using mirVana™ miRNA isolation kit (Ambion, Austin, TX) following the manufacturer’s instructions. The expression levels of mature miR-7 and relative miRNAs were detected by qRT-PCR. A cDNA library was generated using M-MLV reverse transcriptase (Promega, Madison, WI) with specific RT-PCR primers. qPCR was performed using SYBR Premix EX Taq (TaKaRa, Otsu, Japan). The expression of miRNAs or mRNA was quantified by RT-PCR using 7500 Fast Real-Time PCR System (Applied Biosystems), and was normalized to U6B small nuclear RNA (RNU6B) or GAPDH. All samples were prepared and analyzed three times individually. Primers are as follows, GAPDH-F/R: 5′-TGAGTCAACACCTACCCAGCTCCAG-3′/5′-CAGAGTCAGTGATGGGGGGCTTG-3′; KLF4-F/R: 5′-GTCGGATTGAAGTGCTGAGC-3′/5′-ATCGTCTCTCTTCCCTTGGC-3′.

### Western blot analysis of KLF-4 expression

Western blot assays were performed as described previously [10]. Briefly, cells were lysed on ice in RIPA buffer (150 mM NaCl, 50 mM Tris pH 7.5, 1% TritonX-100, 5 mM ethylenediaminetet–raacetic acid). After extraction of total protein, BCA Kit (Pierce, IL, USA) was used for protein concentration detection. Samples (30 μg) were separated by sodium dodecyl sulfate (SDS)-polyacrylamide gel electrophoresis (PAGE) and transferred onto nitrocellulose (NC) membranes. Membranes were incubated with primary antibody KLF-4 (Santa Cruz Biotech, Santa Cruz, CA) at 4 °C overnight, followed by secondary antibody (Santa Cruz Biotech, Santa Cruz, CA). GAPDH (Cell Signaling Technology, Boston, USA) was used as a loading control. The Fusion FX7 system (VilberLourmat, France) was used for protein band visualization.

### Luciferase reporter assay

In order to check the relationship between miR-7 and KLF-4, fragments of 3′-UTR (Wt) containing the binding site of miR-7, or 3′-UTR mutant (Mut) of KLF-4 were cloned into the pMIR-Report luciferase vector. SMMC-7221 cells were cotransfected with luciferase reporter vector, Renilla luciferase control vector (pRL-hTK) and miR-7 mimics or the negative control miRNA, using Lipofectamine 2000 (Invitrogen). Luciferase assays were performed 48 h post-transfection using the dual-luciferase reporter assay system (Promega). Firefly luciferase activity was normalized to Renilla luciferase activity.

### MTT assay

24 h after transfection, Hep3B and SMMC-7221 cells were seeded into 96-well plates. The viability of the cells were evaluated by 3-(4, 5-dimethylthiazol-2-yl)-2, 5-diphenyl-tetrazolium bromide (MTT) assays according to the literature [15].

### Matrigel invasion assay

50,000 labeled cells (using Cell tracker green, Invitrogen) were seeded into Matrigel-coated Transwell insert (Corning) in DMEM with 10% serum. The bottom side of transwell was filled with DMEM containing 20% serum. After 24 h, labeled cells were counted under a fluorescent microscope.

### Statistical analysis

SPSS software (version 20.0) was used for all statistical calculations. Values were presented as mean ± standard deviation (SD). All experiments were repeated at least three times, and comparisons between two means were determined by the paired Student’s *t* test. Statistical significance was established at *P* value less than 0.05.

## Results

### MiR-7 is downregulated in HCC tissues

**Fig. 1:**
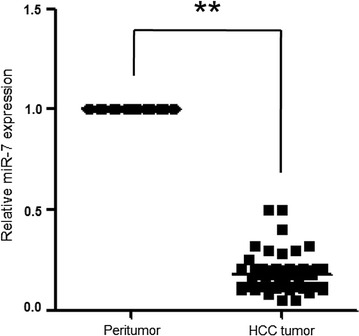
Analysis of miR-7 expression in hepatocellular carcinoma (HCC) tissues and matched peritumor tissues by qRT-PCR. HCC tumor tissues displayed significantly lower miR-7 expression level than the matched peritumor tissues. **P < 0.01 vs. peritumor tissues

To evaluate whether miR-7 is aberrantly expressed in HCC tumor tissues, the expression of miR-7 in HCC tumor tissues and matched peritumor tissues obtained from 50 patients was determined using qRT-PCR. The miR-7 expression in tumor tissues is significantly lower than that of the matched control (0.18 ± 0.04 vs. 0.95 ± 0.07, *P* = 0.0028, Fig. 1), indicating that miR-7 may be a tumor suppressor in HCC. The miR-7 expression levels were classified to either low or high according to the median value of the cohort. The statistical analysis revealed a significant association between the low expression of miR-7 and tumour size (*P* = 0.0124) (Table 1).

**Table 1:** Association between miR-7 expression and clinicopathologic characteristics

Features	n (%)	High expression (%)	Low expression (%)	*P*/χ^2^ value
Gender				0.879/0.023
Male	27 (77.6)	13 (67.8)	14 (32.2)	
Female	23 (22.4)	10 (45.7)	13 (54.3)	
Age (year)				0.653/0.365
≥60	23 (62.2)	14 (60.8)	9 (39.2)	
<60	27 (37.8)	13 (55.9)	14 (44.1)	
Tumor size (mm)				0.012/10.054
≥50	28 (71.8)	8 (71.4)	20 (28.6)	
<50	22 (28.2)	14 (81.8)	8 (18.2)	
AFP (ng/mL)				0.275/1.256
>400	23 (49.4)	9 (45.5)	14 (54.5)	
<400	27 (50.6)	13 (40.5)	14 (59.5)	
TNM stage				0.027/12.864
T1	16 (35.9)	11 (39.3)	5 (60.7)	
T2/T3	34 (64.1)	16 (46.0)	18 (54.0)	
Intrahepatic metastasis status				0.019/1.358
Positive	15 (30.0)	12 (80.0)	3 (20.0)	
Negative	35 (70.0)	18 (51.4)	17 (48.6)	
Tumor number				
Single	32 (64.0)	14 (43.8)	18 (56.2)	0.504/9.281
Two or more	18 (36.0)	8 (44.4)	10 (55.6)	

The statistical significance of the individuals was determined with χ^2^ test. *P* < 0.05 was considered significant

### MiR-7 inhibits the proliferation and invasion of HCC cells

**Fig. 2:**
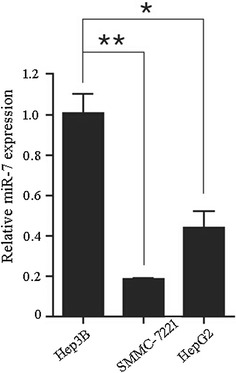
Analysis of miR-7 expression levels in different HCC cell lines by qRT-PCR

In order to check the inhibitory effect of miR-7 on cell proliferation and invasion of HCC cells, we first determined the expression level of miR-7 in HepG2, Hep3B and SMMC-7221 cell lines by qRT-PCR. As shown in Fig. 2, Hep3B cells displayed significantly higher miR-7 expression level as compared with SMMC-7221 and HepG2 cells.

**Fig. 3:**
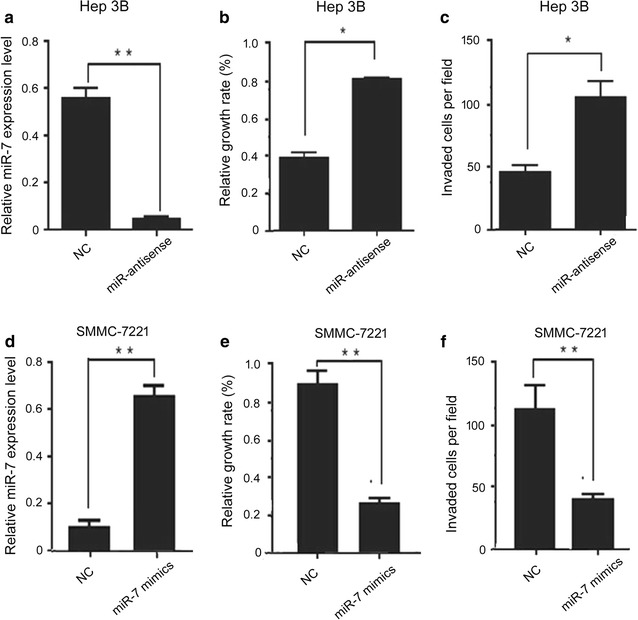
Cell proliferation and invasion of HCC cells detected by MTT assay and matrigel invasion assay, respectively. **a** Expression levels of miR-7 were detected in Hep3B cells transfected with miR-7 antisense or NCi; **b** relative growth rate of Hep3B cells transfected with miR-7 antisense or NCi; **c** invaded cells per field Hep3B cells transfected with miR-7 antisense or NCi; **d** expression levels of miR-7 were detected in SMMC-7221 cells transfected with miR-7 mimics or NCi; **e** relative growth rate of SMMC-7221 cells transfected with miR-7 antisense or NCi; **f** invaded cells per field in SMMC-7221 cells transfected with miR-7 antisense or NC

For comparison, overexpression of miR-7 mimics of SMMC-7221 cells and overexpression of miR-7 antagomirs Hep3B cells were established. After transfecting miR-7-antisense into Hep3B cells, the miR-7 expression level was dramatic decreased compared with the negative control (treated with the same length of RNA, without any bioactivity) (Fig. 3a). The results from MTT assay showed that the miR-7-antisense-transfected Hep3B cells exhibited faster cell growth rate than the control cells (Fig. 3b). The matrigel invasion assay showed a higher invasive rate in miR-7-antisense-transfected Hep3B cells compared with the control cells (Fig. 3c). In contrast, SMMC-7221 transfected with miR-7 mimics showed an increased miR-7 expression (Fig. 3d) and a decreased cell growth (Fig. 3e) and invasion (Fig. 3f), compared with the control cells. Together, these results suggested that miR-7 inhibits cell proliferation and invasion of HCC cells.

### The interaction between KLF-4 and miR-7 in HCC

**Fig. 4:**
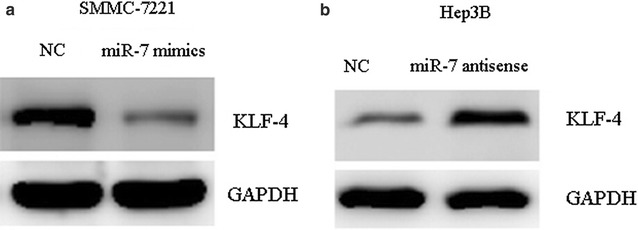
Expression of KLF-4 protein level in miR-7 silenced Hep3B cells detected by Western blot. **a** Expression level of miR-7 was detected in SMMC-7221 cells transfected with miR-7 mimics or NC; **b** expression level of KLF-4 protein was detected in Hep3B cells transfected with miR-7 antisense or NC

**Fig. 5:**
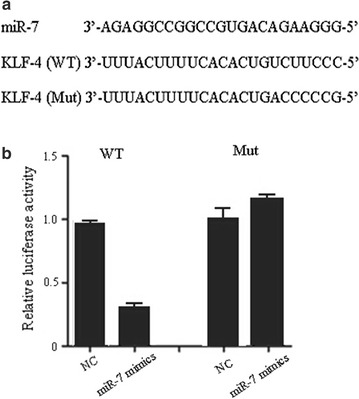
Direct target of miR-7 in HCC cells. **a** Putative miR-7 binding site in the 3′-UTR of KLF-4 gene; **b** HCC cell lines were transfected with wild-type (WT) or mutant (Mut) 3′-UTR-reporter constructs together with miR-7 mimics or negative control (NC) and the luciferase activity was detected

**Fig. 6:**
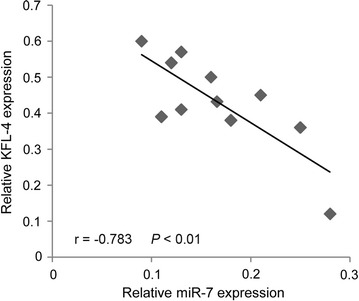
The expression of miR-7 is inversely correlated with KLF-4 expression in 10 HCC samples. qRT-PCR was performed to determine the expression of miR-7 and KLF-4 in HCC sample. r = −0.783; *P* < 0.01

MiRNAs function via suppressing or degrading the segments mRNA of target genes. In order to indentify the target genes interacted with miR-7, TargetScan 7.0 was employed for the prediction. A list of candidate targets of miR-7 were obtained, and among them, KLF-4 appears to be a promising target as it is implicated in the pathology of cancer. Therefore, we the protein level of KLF-4 in miR-7-silenced Hep3B cells. The results showed that the expression level of KLF-4 was significantly decreased in SMMC-7221 cells transfected with miR-7 mimics (Fig. 4a), whereas KLF-4 protein expression level in the miR-7-silenced Hep3B cells was significantly increased compared with the control cells (Fig. 4b). These results indicated that KLF-4 is a potential target of miR-7. To further validate this assumption, luciferase reporter assay was performed to analyze the relationship between KLF-4 and miR-7 in HCC cells. The results showed that miR-7 mimics significantly decreased the luciferase activity of KLF-4 3′-UTR, but had little effect on KLF-4 3′-UTR mutant (Fig. 5b), confirming that KLF-4 is a target of miR-7. Moreover, we observed a statistically significant inversed correlation between miR-7 and KLF-4 in HCC samples (Fig. 6).

### MiR-7 regulates KFL-4/PI3K/Akt pathway

**Fig. 7:**
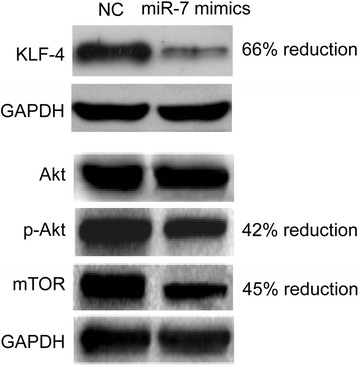
Western blot analysis of protein expression level of KLF-4 and the key components of the PI3K/Akt pathway in miR-7 overexpressing SMMC-7221 cells

PI3K/Akt pathway plays critical roles in tumorigenesis and cancer development. Therefore, we sought to examine if miR-7 has impact on this pathway. In miR-7 overexpressing SMMC-7221 cells, we observed a significant decrease of KLF-4 expression, companied with a significant reduction of AKT phosphorylation and mTOR expression (Fig. 7). This result indicated that miR-7 regulates the KFL4/PI3K/Akt pathway.

## Discussion

In this study, we found that miR-7 was downregulated in clinical samples, and the low expression of miR-7 showed a significant association with tumor size, suggesting an importance role of miR-7 in HCC tumorigenesis.

Targets of cancer-related miRNAs and their roles in tumorigenesis are needed to be fully understood [16]. Recently, it was reported that there were two ways for miRNAs to work. One way is to prevent translation, and the other one is to promote the degradation of specific target mRNAs by binding to their 3′ UTRs [17]. However, a novel assortment of research has demonstrated that miRNAs can function to post-transcriptionally stimulate gene expression through direct mechanisms, and this process is termed “activation” [18]. MiR-7 is highly expressed in parts of the brain, eyes, and pancreas, suggesting its role in the development of these organs. Studies showed that in glioblastomas and breast cancer, the expression of miR-7 was aberrant. Many potential targets of miR-7 were reported to participate in some important signalling of tumor progression, such as targets EGFR, IRS-1, PAK-1, RAF-1, SATB1, and so on [11, 19]. In this study, results showed that KLF4 was a critical downstream target of miR-7 in HCC. Moreover, we found a predicted miR-7 binding site on the KLF-4 transcript and results from the luciferase reporter assay confirmed the interaction between miR-7 and KLF-4. Meanwhile, results of Western blot demonstrated that miR-7 negatively regulated KLF-4 expression. Previous report has shown that miR-7 abrogates KLF4/PI3K/Akt pathway and inhibits prostate tumorigenesis [20]. In this study, we also found miR-7 could inhibit cell proliferation and invasion of HCC. Moreover, overexpression of miR-7 significantly suppressed the expression of KLF4, as well as the phosphorylation and expression of PI3K/Akt pathway downstream effectors.

## Conclusions

In summary, we revealed a close relationship between miR-7 and KLF-4. Our results showed that the low expression of miR-7 was significantly associated with tumour size, indicating that it might serve as an independent prognostic factor for HCC disease. We also identified that KLF-4 is a target of miR-7 in HCC cells. This newly identified target KLF-4 of miR-7 may be considered as a potential prognostic marker and a therapeutic target for HCC patients.

## Abbreviations

HCC: hepatocellular carcinoma
KLF-4: Kruppel-like factor 4
IRS1: insulin receptor substrate 1
EGFR: epidermal growth factor receptor
PAK1: p21 protein (Cdc42/Rac)-activated kinase 1
FBS: fetal bovine serum
RNU6B: U6B small nuclear RNA
EGFP: enhanced green fluorescence protein
RFP: red fluorescent protein
